# Combinatorial optimization by weight annealing in memristive hopfield networks

**DOI:** 10.1038/s41598-020-78944-5

**Published:** 2021-08-12

**Authors:** Z. Fahimi, M. R. Mahmoodi, H. Nili, Valentin Polishchuk, D. B. Strukov

**Affiliations:** 1 UC Santa Barbara, Santa Barbara, CA 93106-9560 USA; 2 Linkoping University, 60174 Norrköping, Sweden

**Keywords:** Nanoscale devices, Electrical and electronic engineering

## Abstract

The increasing utility of specialized circuits and growing applications of optimization call for the development of efficient hardware accelerator for solving optimization problems. Hopfield neural network is a promising approach for solving combinatorial optimization problems due to the recent demonstrations of efficient mixed-signal implementation based on emerging non-volatile memory devices. Such mixed-signal accelerators also enable very efficient implementation of various annealing techniques, which are essential for finding optimal solutions. Here we propose a “weight annealing” approach, whose main idea is to ease convergence to the global minima by keeping the network close to its ground state. This is achieved by initially setting all synaptic weights to zero, thus ensuring a quick transition of the Hopfield network to its trivial global minima state and then gradually introducing weights during the annealing process. The extensive numerical simulations show that our approach leads to a better, on average, solutions for several representative combinatorial problems compared to prior Hopfield neural network solvers with chaotic or stochastic annealing. As a proof of concept, a 13-node graph partitioning problem and a 7-node maximum-weight independent set problem are solved experimentally using mixed-signal circuits based on, correspondingly, a 20 × 20 analog-grade TiO_2_ memristive crossbar and a 12 × 10 eFlash memory array.

## Introduction

Combinational optimization is an essential subset of mathematical optimization methods with numerous applications in various fields, including operation research, machine learning, and scientific computing^1–3^. A typical goal of combinatorial optimization is to find an optimal solution within a finite set of possible solutions. For example, graph partitioning, that is, the problem of minimizing the cutsize when partitioning a graph into two sections of nearly equal weight, finds applications in distributed computing and digital VLSI design flow.

For most combinatorial problems, the exhaustive brute-force search is often not practical, and developing efficient heuristic and meta-heuristic methods is of utmost importance^4,5^. The enormous computational power required to solve large-scale optimization problems also poses a great challenge. The problem is exacerbated by the sequential structure of general-purpose processors, which are very energy-demanding and inefficient in running large-scale, massively parallel algorithms. Hence, hardware accelerators, e.g., based on superconductors^6,7^, digital CMOS^5,8,9^, nanomagnetic^10^, and photonic ^11^ technologies, are proposed to solve optimization problems using heuristic methods efficiently. Hopfield neural network (HNN)^12–14^ is also a heuristic method that extended the application of neural networks from classification to optimization and associative memory. A particular class of recurrent HNNs is the discrete-time asynchronous model, which operates based on a single neuron update at a time mechanism. For a network featuring N binary neurons, a randomly-selected *j*th neuron is updated at time *t* + 1 using

1 $$ \begin{array}{*{20}c} {U_{j} \left( {t + 1} \right) = f\left( {\mathop \sum \limits_{i = 1}^{N} w_{ij} \left( t \right)U_{i} \left( t \right) + T_{j}^{b} } \right),} \\ \end{array} $$

where *U*_*j*_(*t*) is the binary state of the *j*th neuron at time *t*, *w*_*ij*_(*t*) is the synaptic strength between neurons *i* and *j* at iteration *t*, $T_{j}^{b}$ is the bias strength of the *j*th neuron, and f(.) is the binary threshold function. The key features of HNNs are their activation dynamics and energy function, which are proven to be monotonically descending during the runtime^15^ (see Supplementary Section 1 for more details). Hence, by mapping the cost function of the optimization problem into the energy of the network and the variables to neuron states, the recurrent dynamic of the network optimizes the cost function and solves the optimization problem in the runtime.

Similar to the Ising model and other greedy and local search methods, the critical shortcoming of HNN is the presence of (many) local minima in their energy function. Simulated^16,17^ and chaotic^18–20^ annealing are two prominent techniques that tackle this issue by harnessing thermally controlled probabilistic jumps and embedded chaos in HNNs with nonzero self-feedback weights, respectively. Therefore, an efficient HNN accelerator should perform the frequent dot-product operation in Eq. 1 very fast and support an annealing technique to rescue the network from trapping in local minima. This paper introduces a weight annealing technique in HNNs and its efficient implementation, which is more effective and scalable than simulated and chaotic annealing methods. Our approach dates back to methods like weight annealing^21–24^, noising^25^, space smoothing^26,27^, and fine-tuned learning^28^, where the core idea is to change in the energy landscape by modifying weights in the formula for the energy. Here, the exact meaning of “weight” varies from method to method, as well as from problem to problem addressed—a weight may be associated with an input data point, a subproblem, etc.; similarly, a variety of ways to modify the weights (random perturbation, adversarial change, etc.) has been explored. The common crux of the methods is that they modify the weights differently in every timestep and in different areas of the solution space; this way, the search is guided by weight changes adapted to the current state and reuses insights gained from previous iterations. While the clever schemes for such adaptive weight modifications underpin the strengths of methods, mimicking this adaptivity within any hardware would likely be inefficient since performing individual changes to the weights consumes significant time and energy. Further, hardware implementation of the algorithms that act differently in different parts of the solution space would require complicated circuitry, leading to efficiency losses. Our proposed weight annealing circumvents both of the above: First, all weights are scaled together at every iteration. Second, the weight modification is oblivious to the status of the solution space exploration—the annealing schedule is pre-set in advance and does not depend on the state of the system (in particular, the schedule does not depend on the value of the energy function—it is the hardware that takes care of the derivatives, convergence, escaping local optima with stochastic decisions, etc.). We numerically demonstrate the effectiveness of our approach on several benchmarks by solving graph partitioning, vertex cover, maximum-weight independent set, and maximum-weight clique problems.

We also propose a very efficient implementation of weight annealing in HNNs harnessing analog-grade non-volatile memories, which have become the mainstream devices for implementing fast, compact, and energy-efficient dot-product engines^29–32^. The potentials for performing high-speed physical-level computing are perhaps the most intriguing feature of these devices. Passive (0T1R) memristive devices are the most promising candidate for the next generation of analog computing systems in part due to their excellent scalability prospects and superior integration density^33–36^. Furthermore, recent breakthroughs in exploiting embedded eFlash memories have opened the doors towards building large-scale industrial-grade neurocomputing systems^32,37^ as well. These exciting opportunities have served as the motivations for several experimental proposals on Hopfield networks, simulated annealing, and related concepts.

Reference^38^ uses discrete Pt/TiO_2−x_/Pt memristive devices to implement a small-scale 4-bit data converter with the Hopfield model. Reference^39^ implements a 3-bit associative memory based on digital HfO_2_ memristors. In Ref.^40^, simulation results demonstrate the effectiveness of using the inherent chaos in sub-100 nm NbO_2_ memristors to implement simulated annealing within Hopfield networks. Ref. ^41^ implements an 18-node restricted Boltzmann machine based on a versatile stochastic dot-product engine using TiO_2_ memristive crossbars^42^. In addition, Ref. ^41^ demonstrates hardware implementation of simulated, chaotic, and adjustable annealing within HNNs. Conceptually, the proposed weight annealing is similar to the adjustable technique as it relies on dynamic scaling of the energy during runtime. However, the proposed method has a more straightforward implementation as it does not require extra circuitry, is not limited to the dynamic range of devices, and can be generally applied to any HNN irrespective of the target optimization problem. Several works (e.g., see^43–45^) propose using the inherently random switching mechanism of memories to implement stochastic sigmoid neuron functionality and simulated annealing. However, this method suffers from the limited switching endurance, cycle-to-cycle and device-to-device variations, and scalability issues. Finally, Ref. ^46^ uses Y-flash memories to implement a 3-bit associative memory based on the Hopfield model.

## Results

The proposed idea is to slowly modulate the energy landscape of the HNN, starting from a funnel shape with a deep global optimum where the ground state is easily accessible. The network traps in it in the early stages and tends to remain in ground states during the runtime. In our proposed method, we change the synaptic weights slowly by considering $w_{ij} = T_{ij} \left( {1 - e^{{\frac{ - t}{\tau }}} } \right)$ where $\tau$ > 0 is the annealing schedule, and $T$ is the ultimate synaptic weight matrix. The Lyapunov energy associated with a certain state of the network at $t$ is given by

2 $$ {E\left( t \right) = - \frac{1}{2}\mathop \sum \limits_{i = 1}^{N} \mathop \sum \limits_{j = 1}^{N} w_{ij} \left( t \right)U_{i} \left( t \right)U_{j} \left( t \right) - \mathop \sum \limits_{i = 1}^{N} T_{j}^{{\text{b}}} U_{j} \left( t \right).}  $$

At the beginning and while t<<τ is very small, the first term [in Eq. (2) ] is negligible, and the total energy of the network is $- \sum\nolimits_{i = 1}^{N} {T_{j}^{b} U_{j} \left( t \right)}$. At this stage, the network finds a straightforward solution after few updates. The ground state, for example, is located at $U_{j} = 1$ for the *j*th neuron that has $T_{j}^{{\text{b}}} > 0$. As the network evolves, $w_{ij} \left( t \right)$ gradually moves toward $T_{ij} \left( t \right)$ and the first term in Eq. (2) becomes more significant until the network stabilizes in the equilibrium state. During this runtime, the ground state of the network changes many times, but the network tends to capture it and closely follows the transitory ground state.

**Figure 1:**
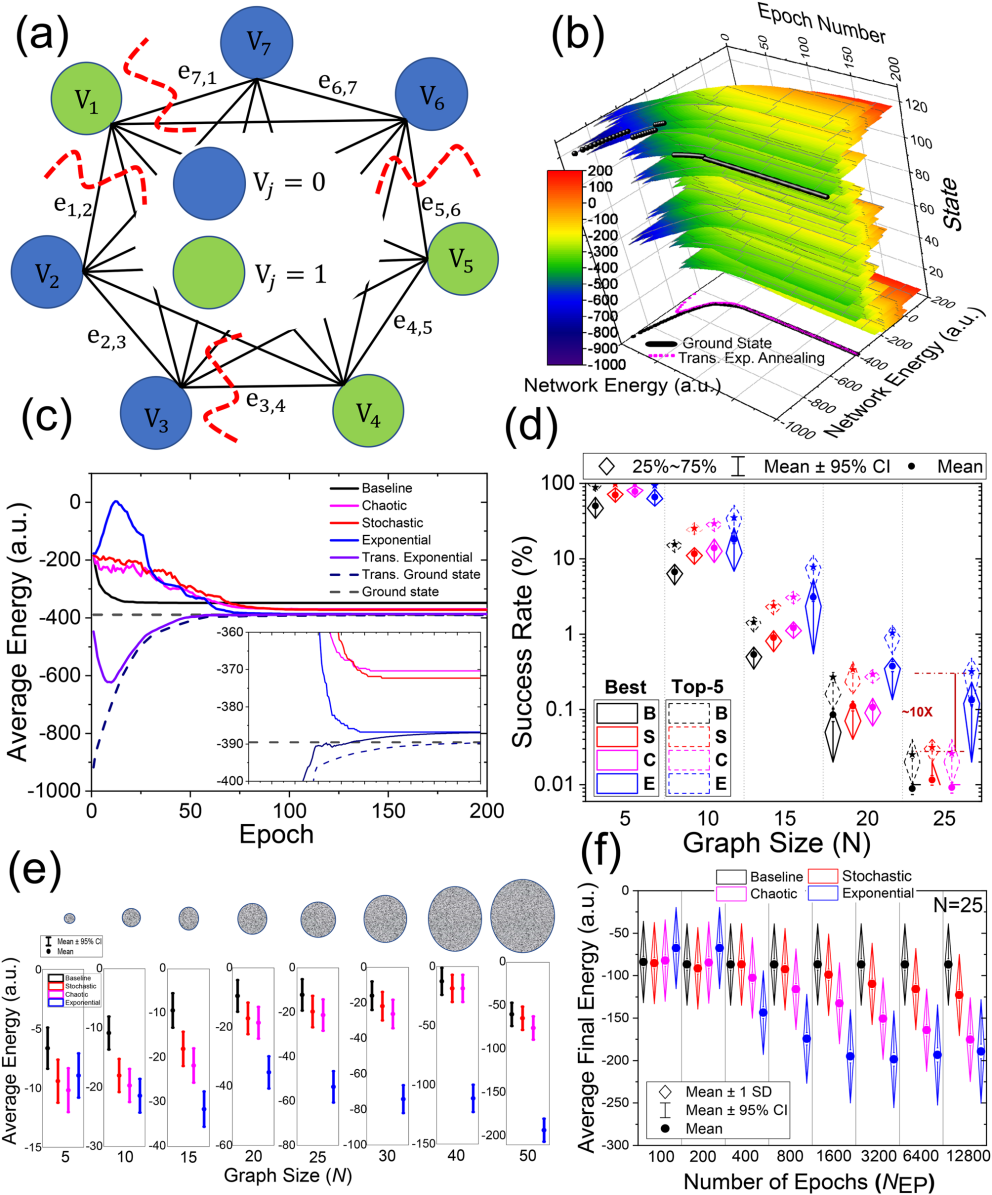
Neuro-optimization with the weight annealing: (**a**) The 7-node weighted graph partitioning problem that is used to illustrate the mechanism of weight annealing. The blue/green coloring shows the optimum solution (Supplementary Materials S3 includes the actual weights). (**b**) The energy evolution of each state during weight annealing for 200 epochs and $\tau = 40$. The black spheres mark the transitory ground state of the system, which is also projected to the energy-epoch plane. The magenta curve shows the average transitory energy over 128 runs, which shows that the proposed weight annealing tracks the transitory globally optimum state of the system. (**c**) The average energy of the network annealed with different techniques over 128 runs. (**d**) Top-1 and Top-5 success rates of varying annealing techniques versus problem size (B: baseline, i.e., the standard Hopfield network without annealing, S: stochastic (temperature reduced from 100 to 0.01), C: chaotic (temperature reduced from 250 to 0.001), and E: exponential weight annealing). For each graph size, we consider 200 randomly weighted problems and provide the parameters in supplementary S4. Note that the best response is the global optimum, and Top-5 counts if the final response is among the best top-5 solutions. Panel (**e**) shows the distribution of the final average energy, offset by a constant for clarity, for the same graphs used in panel (**d**). The circles represent graph size. (**f**) The boxplot of the average final energy vs. epoch size for 200 random configurations of 25-node graph partitioning problems.

We consider graph partitioning problems (see Supplementary Section 2) that find applications, e.g., in graph-based electronic structure theory applied to quantum molecular dynamic simulations^45^. To demonstrate a clear visual representation of ground state evolution, we use a 7-node graph partitioning problem with randomly selected weights and edges, as shown in Fig. 1a (see Supplementary Sect. 3 for the actual vertex and edge weights). Figure 1b shows the semi-exponential energy change of all possible states during the annealing ($\tau = 40$). The energy associated with each state is exponentially increasing as expected. The black sphere points (projected to the bottom plane for clarity) represent the ground state of the system during the annealing. The global optimum is − 389.5459 and locates at state 97 (decimal equivalent of “1,100,001”). The transitory state of the system is specified by listing the $N$ values of $U_{j}$ and represented by a binary word of $N$ bits^14^ and its decimal version for simplicity. At $t = 0$, the global minimum is recognizable (state 118, *E* = −917.76). While the network is steadily evolving, the ground state of the system increases, and its location changes several times. The average transitory energy of the system (defined over the transitory synaptic weights) is also shown for 128 initialization schemes and 200 epochs ($N_{{{\text{EP}}}} = 200$) in magenta. The network finds the initial ground state very quickly (regardless of the initial state) owing to the annealing mechanism and tracks it during the evolution. Other simulation details, including $w_{ij}$ evolution are provided in Supplementary Sect. 3.

Figure 1c shows the performance of the proposed annealing technique versus stochastic annealing with a probabilistic sigmoid neuron (the temperature is reduced exponentially from 100 to 0.01) and chaotic annealing (the self-feedback weights are decreased exponentially from 250 to 0.001). In this experiment and after 200 epochs, the success rate (the relative number of cases led to the global optima) is 57.8%, 59.37%, 94.53% for chaotic, stochastic, and weight annealing techniques, respectively, and it is 28.12% for the standard Hopfield model (baseline). It is noteworthy that the stochastic annealed network converges to E = −387.98 and scores a 98.6% success rate when 30 k epochs are used, and the temperature is scaled from 100 k to 0.01.

To further investigate the performance of the proposed approach, 200 randomly populated configurations of 5, 10, 15, 20, and 25-node graphs are considered. Supplementary Sect. 4 discusses the parameters used in the simulations. The annealing schedule parameter is manually optimized for the first problem and used in all configurations. The scalability of our approach is compared with simulated annealing on three scenarios: first, $N_{{{\text{EP}}}} = 300$ is assumed for all sizes, then it is exponentially increased for a fixed-size graph ($N$ = 25), and then, $N_{{{\text{EP}}}}$ is exponentially increased with respect to the linear increase of the problem size.

The success rate achieved by different methods on various problem sizes for $N_{{{\text{EP}}}} = 300$ is shown in Fig. 1d. The performance of weight annealing is on par with simulated annealing for *N* = 5; however, the energy gap becomes significantly wider for larger problem sizes. More interestingly, for *N* = 15, among the 200 configurations, the 20 percentiles success rate of weight annealing is better than the 80 percentiles of all other methods. Note that due to the analog-grade behavior of our memristors, weighted graph problems are considered, and it would be unfair to compare our results (in terms of success rate) with previous implementations, which focus on sparse graphs with binary weights. Figure 1e shows the average final energy for the same graphs. The gap between the solution quality (final energy) of exponential weight annealing and other methods becomes wider in more massive graphs. In Fig. 1f, the computational runtime (epoch number) is increased for 200 configurations of 25-node graphs. As expected, the performance of all annealing techniques, including weight annealing, improves by increasing the number of epochs (in part due to slower cooling, which allows the networks to search for better solutions). The performance of weight annealing no longer improves for *N*_EP_ > 3200, while simulated annealing techniques, with noticeable inferior performance, benefit from the longer computational time and slower annealing. This is partly due to the inherent differences between the underlying mechanism of simulated and exponential weight annealing. Stochastic annealing requires more time to explore larger searching spaces. While for the weight annealing, it is simply not the case. The accuracy saturation stems from the fact that the slower learning of weights no longer creates a more optimum path. Note that weight annealing achieves the same solution quality 10 × faster than simulated annealing techniques. Supplementary Sect. 4 extends the graph partitioning simulations. Three other combinatorial optimization problems are considered in Supplementary Sect. 5, and the results signify the superiority of weight annealing, particularly in large scale problems.

## Experimental results

The proposed technique is demonstrated by addressing two optimization problems based on the most prospective analog-grade memory technologies. The central merit of weight annealing lies in its very straightforward and compact implementation. Experimental results of hardware implementation are demonstrated by solving a 16-node graph partitioning problem using a 20 × 20 passively integrated analog-grade memristive crossbar and a 7-node maximum-weighted independent set on a 12 × 10 embedded array of eFlash memories.

**Figure 2:**
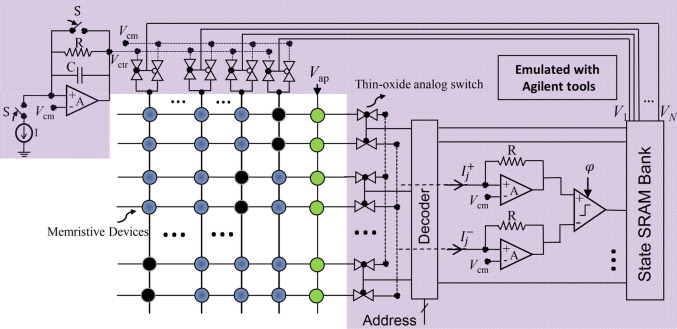
The current-mode recurrent circuit that implements the weight annealing of discrete-time Hopfield networks with programmable analog memories. The green circles show the bias weights ($$T_{i}^{{\text{b}}}$$) while the black circles implement self-feedback weights ($$T_{ii}$$), and the rest of them denote the main synaptic weights ($$T_{ij,i \ne j}$$). A constant ‘on’ voltage, which is the same as the tuning voltage, drives the bias column. We control the applied voltage to the rest of the devices during the runtime to adjust the synaptic weights (exponentially). Note that $$V_{{{\text{cm}}}}$$ is only added to emphasize that the circuit operate on a single-$$V_{{{\text{dd}}}}$$. Values $$R, C, $$ and $$I$$ depend on the problem size and technology, and determine the annealing schedule. Switch S resets the network to the initial condition. The selected neuron is determined by the input address to the decoder, and the operation is synchronized with the sampling clock ($$\varphi$$) in dynamic comparator. Note that we have omitted the tuning circuits in the figure for clarity.

Figure 2 shows the implementation of the weight annealing technique. The corresponding hardware realization of Eq. (1) is discussed in the method section for both cases. The main challenge in realizing the weight annealing is scaling the synaptic weights. Let us emphasize that direct modification of (analog) states is impractical in part because of the limited endurance, device-to-device, and cycle-to-cycle variations. This challenge can be resolved in resistive memories by using a simple control circuit (the pre-synaptic drivers), which scales all synaptic weights simultaneously (see Fig. 2). Here, $V_{{{\text{ctrl}}}}$ is exponentially increased toward $V_{{{\text{ap}}}}$ at which all devices are tuned. The current neuron state determines which devices should be driven by $V_{{{\text{ctrl}}}}$. The post-synaptic circuits include trivial circuits such as transimpedance amplifiers (e.g., a buffered version of Ref.^47^) that senses currents and a dynamic voltage comparator (see, e.g.,^48^) that updates the selected neuron state. These circuit functionalities are emulated with Agilent characterization tools in the present demonstration.

In split-gate embedded Flash memories, the situation is more straightforward as we can bias the memories in the weak inversion regime, making their states (i.e., currents) semi-exponentially dependent on the select-gate voltage. Then, $V_{{{\text{ctrl}}}}$ is applied to the shared select-gates and linearly increased toward the $V_{{{\text{ap}}}}$.

**Figure 3:**
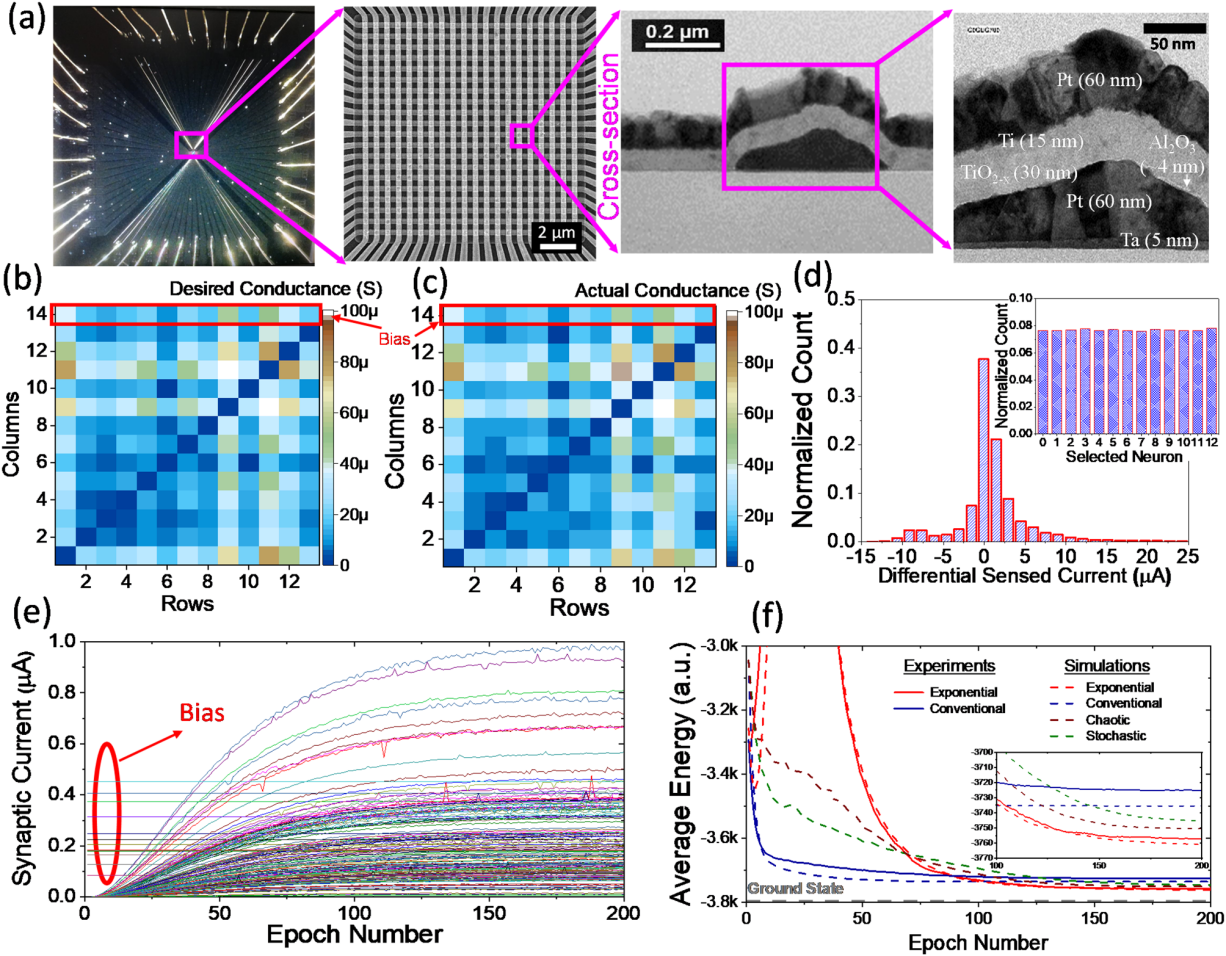
The experimental demonstration with the integrated memristor crossbars. (**a**) The fabricated 20 × 20 integrated memristor crossbar^29^. (**b**) The desired ideal analog map for the 13-node graph partitioning problem, and (**c**) the resultant conductance map of the devices after tuning the crossbar. (**d**) Distribution of the readout current when solving the problem with the conventional (baseline) approach. The inset shows the histogram of selected neurons (for updates) and indicates there is no bias in the neuron update. (**e**) The evolution of the synaptic weights during the weight annealing. (**f**) The experimental versus simulation results of the neuro-optimization with different techniques. The inset shows the zoomed-in average energy in the last 100 epochs.

In the first experiment, a 13-node graph partitioning problem is implemented using passively-integrated memristive crossbars. Note that, to the best of our knowledge, this work is the largest Hopfield network implemented with passive memristors. Figure 3a shows the wire-bonded chip, crossbar TEM image, and an SEM image of a memristive device. This crossbar has been previously used for the demonstration of a multilayer perceptron^49^, integrated spiking neural network for coincident detection^29^, and a hardware security primitive design^50,51^. The method section includes a brief description of fabrication steps. More relevant details are also available in our previous work^49^.

In order to increase the demo size and without the loss of generality, we have ensured the weights and edges (of the graph) are selected such that $T_{ij} < 0$ and $b_{j} > 0$ (see Supplementary Sect. 6 for more details). This facilitates a single-ended time-multiplexed dot-product of a 13 × (13 + 1) network on our memristive crossbars. The details of forming, tuning, and operation of the circuit, as well as the procedure of mapping the actual synaptic weights (from software) to conductance values, are illustrated in the method section. After determining the desired conductance map, the devices are programmed individually using the write-verify algorithm^54^. Figure 3b,c show the desired weight map of the network and the corresponding conductance map obtained after tuning the crossbar, respectively. Most devices are tuned very close (within 5%) to the desired states, which is possible due to the tight distribution of switching thresholds in our analog-grade crossbar circuits. Figure 3d shows the distribution of pre-activation readout currents for the baseline case (the inset indicates no bias in neuron selection). The input “on” voltage corresponding to binary input ‘1’ is *V*_ap_ = 0.1 V. Note that we exponentially increase the “on” applied voltage from 0 to 0.1 V for the weight annealing. The measured synaptic strength of each device during the weight annealing is shown in Fig. 3e. The experimental and simulation results are compared in Fig. 3f. Specifically, the average energy over 10^3^ cases for 200 epochs is shown for various methods. Here, the annealing schedule parameters are 10^4^, 10^5^, and 35 for chaotic, stochastic, and weight annealing, respectively. The ground state locates at − 3796, and weight annealing (on both experiment and simulation) performs better than other techniques and far better than the baseline.

**Figure 4:**
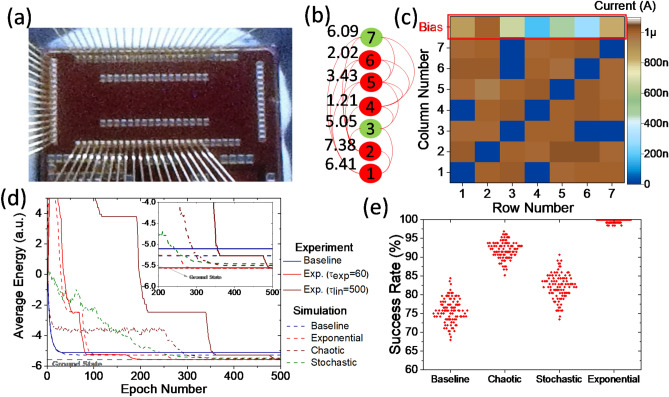
Neuro-optimization with embedded analog-grade eFlash memories. Panel (**a**) shows the fabricated 10 × 12 eFlash array chip in Global Foundries’ standard LPe CMOS process^52^. (**b**) A 7-node maximum-weighted independent set problem. (**c**) The heat map of the synaptic weights for the devices that implement the neuron-optimization. (**d**) The average energy versus epoch comparing experimental results with simulations over 100 runs. (**e**) The success rate of different annealing techniques on this problem over 100 runs.

In our second experimental demo, a 7-node maximum-weighted independent set is solved using an array of 12 × 10 redesigned embedded Flash memories fabricated in Global Foundries 55 nm LPe CMOS process (Fig. 4a). The redesigned array structure enables < 1% analog programmability^52^ (see Fig. 6S). The circuit diagram in Fig. 6Sd implements the weight annealing of Hopfield networks with eFlash memories. Biasing conditions (imposed during programming) ensure the subthreshold operation of the devices at all operating conditions. Figure 4b shows the implemented weighted graph. Similar to the first demo, the weights and edges (of the graph) are chosen randomly but constrained by $T_{ij} < 0$ and $T_{j}^{{\text{b}}} > 0$. The original weight matrix is shown in Supplementary Sect. 7. The ground state of the energy function locates at -5.5755 that corresponds to the neural state “0010001”.

The devices are programmed with < 1% accuracy (see the method section). Figure 4c shows the resultant map of state currents under nominal biasing conditions, i.e., ($V_{{{\text{WL}}}} = 1.5 {\text{V}}$, $V_{{{\text{CG}}}} = 2.5 {\text{V}}$, $V_{{{\text{BL}}}} = 1 {\text{V}},{ }V_{{{\text{SL}}}} = 0 {\text{V}},{\text{ and }}V_{{{\text{EG}}}} = 0 {\text{V}}$). The experiments and simulations are performed over 128 initialization cases for 500 epochs and show the results in Fig. 4d. The results are averaged over 100 runs in the simulations. The annealing schedule is 10, 10, and 100, and the average probability of hitting the global optimum is 0.76, 0.92, 0.82, and 0.99 for stochastic, chaotic, and weight annealing, respectively (Fig. 4e). We drive the devices corresponding to bias weights $(T_{j}^{{\text{b}}}$) by constant gate-voltages ($V_{{{\text{WL}}}} = 1.5 {\text{V}}$ and $V_{{{\text{CG}}}} = 2.5 {\text{V}}$), while other rows (if their corresponding neuron is in the 'on' state) are driven by $V_{i} \left( t \right)$. The impact of annealing schedule and exponential versus linear voltage scaling are also studied in Fig. 7S. For the former, a slower annealing schedule ($\tau_{{{\text{exp}}}} = 60$) tackles the nonlinearities in the super-exponential dependency of synaptic current to voltage and closely match the trends in the simulations. For the latter case, the slowest annealing process ($\tau_{{{\text{exp}}}} = N_{{{\text{EP}}}} = 500$) leads to the best response.

## Discussion and summary

We have demonstrated weight annealing, a technique that improves the performance of asynchronous Hopfield neuro-optimizer. The weight annealing converges faster and to a better solution within studied runtime as compared to other considered annealing approaches. The scalability of weight annealing (size and computational time) is investigated by solving several combinatorial problems, and its straightforward implementation is demonstrated the using two state-of-the-art analog-grade non-volatile memories.

The passive integrated memristor technology offers the best scaling prospects and low fabrication cost. We have recently developed a 4 K fully CMOS-compatible 0T1R array with excellent switching characteristics^36^. The measured analog characteristics are promising for the development of large-scale neuro-optimization systems. On the other hand, eFlash technology is much sparser, but it is currently commercially available and embedded in standard CMOS processes (down to 28 nm). Our preliminary estimations (see Supplementary Sect. 8 for more details) indicate impressive prospects of using metal-oxide memristors for the hardware implementation of Hopfield networks and weight annealing. Future works focus on the CMOS-integrated design of a weight annealing optimizer, allowing us to perform a rigorous comparison with entirely fabricated annealing machines.

As opposed to most previous works^43–45^ that focus on switching statistics of memristors, our proposed solution offers very infrequent writes, which is justified assuming long runtimes of computationally extensive problems. More importantly, our proposed neuro-optimizer offers analog (> 5 bits with memristive nanodevices and > 6 bits via eFlash technology) weights. This feature is not demonstrated in most previous Ising machines. Unlike quantum computing machines that are susceptible to environmental noise, hard to scale, and must operate at cryogenic temperatures, the proposed circuit is more scalable, and can operate at room temperatures.

In summary, the proposed weight annealing boosts the performance of HNN in solving combinatorial optimization problems. Using extensive simulations on four representative problems, we numerically demonstrate that the proposed method outperforms the conventional Hopfield network (baseline) and challenges the prominent stochastic and chaotic annealing techniques in computational time and accuracy. Then, an efficient, scalable, and fast circuit implementation and experimentally verified based on two memory technologies. Large-scale integrated implementation is demonstrated of weight annealing is a near-term future work.

## Methods

In the first experiment, we demonstrate the weight annealing with a 20 × 20 array of passively integrated crossbars of 600-nm pitch memristive devices (200-nm lines separated by 400-nm gaps) fabricated in the University of California at Santa Barbara's nanofabrication facility. The fabrication and characterization details are discussed in ^29,49^. In summary, we deposit the active bilayer by low-temperature reactive sputtering, evaporate electrodes using oblique angle physical vapor deposition, pattern them by lift-off technique, and then contact them to bonding pads. The crossbar is wire-bonded in a dual in-line package and mounted on a custom-made printed circuit board, as shown in Supplementary Section 9.

The devices are in pristine states upon fabrication and require electroforming to become programmable devices. An automated setup performs the current-controlled electroforming process device per device. A compliance voltage (1.5 V to begin with, but it is dynamically updated) prevents the memristors from burning. For every device, we sweep the applied current from 0 to 100 µA and monitor its resistance consistently. The process continues until the device reaches an acceptable low resistance (typically 5 kΩ–150 kΩ). The devices are formed individually and reset them after each forming success (to remove leakage for the rest of the crossbar). A dynamic leakage removal procedure is also employed to reset the devices when the algorithm struggles to form several devices in a row.

The devices are tuned using an ex-situ approach meaning that weights ($T_{ij}$) and biases ($T_{b}$) are obtained from software simulations and later transferred to the crossbar. Indeed, after forming the entire crossbar, i.e., the 400 devices (yield is typically > 99%), the memristors are tuned to the desired states individually using V/2 and write-verify schemes. The automated algorithm progressively increases the pulse amplitude from 0.5 to 2 V (to increase the conductance) and from 0.5 to 2.2 V (to reduce it). The pulse width is 1.1 ms during the programming. Each device typically needs ~ 50 pulses to reach within 2% of the targeted state. The fabricated crossbar has a reasonably uniform and tightly distributed switching thresholds ranging from 0.6 to 1.5 V (for set) and − 0.6 to − 1.7 V (for reset), which provides us with the opportunity to harness the V/2 scheme and precisely tune the devices. The devices have excellent retention characteristics, and accelerated retention tests report minor < 1% change in after the projected 10 years of operation at room temperature. Additional details are provided in Ref.^22^.

In order to increase our demo size (given our 20 × 20 crossbar size), we deliberately chose edges to be larger than weights (the values are selected randomly in all experiments and simulations) to force all non-diagonal synaptic weights ($T_{ij}$) to be negative and all biases to be positive. This technique allows us to implement a relatively larger demo by assigning one device per weight (in comparison with the two-device per synapse needed for fully differential design) and perform each the vector-by-vector multiplication in two cycles. Indeed, the dot-product operation is implemented in a two-step time-multiplexed fashion; that is, in one cycle, we measure the total current ($\sum I^{ - }$) associated with the input vector multiplied by the synaptic weight vector (from the selected neuron), while the input bias voltage is zero. Then, we subtract it from the sensed current ($\sum I^{ + }$) from the same bitline, while the main inputs are zero and apply $V_{{{\text{ap}}}}$ = 0.1 V to the bias column. Besides, to increase the dynamic range, all bias conductances are divided by 5 and compensated by applying an extra gain of 5 at the neuron side. In other words, the final output is evaluated by hard thresholding $\left( {5\sum I^{ + } - \sum I^{ - } } \right)$. (Note that we have previously fully-differential single-shot dot-product engines are already demonstrated using the same devices in our previous works—see, e.g.,^29,39^), and this simple trick is employed only to enlarge the problem size.

Owing to the single-ended design, we use $g_{ij} = G_{{{\text{max}}}} \left( {T_{ij} /\max \left( {\left| {T_{{{\text{max}}}} } \right|} \right)} \right)$, where $\max \left( {\left| {T_{{{\text{max}}}} } \right|} \right)$ is the maximum absolute weight and $G_{{{\text{max}}}}$ is the maximum absolute conductance (40 µS in our experiment). We ground all bitlines (bottom electrode) except the one associated with the selected neuron, which is virtually grounded, and its current is sensed using a B1530A fast measurement unit and a B1500A parameter analyzer. We apply neuron voltages to the switch matrix, connected to both 20 rows and 20 columns of the crossbar. We link top electrodes to the input neurons and bottom electrodes to the output neurons through an E5250A switch matrix.

The eFlash chip, fabricated in Global Foundries 55 nm LPe process, includes a 12 × 10 redesigned industry-grade split-gate memory array. The packaged chip is previously used for developing a high-performance dot-product engine^52^. Agilent B1500A and B1530A tools are used for measurements and pulse generation. We have developed a custom-made switch matrix on a printed circuit board controlled via a lightweight microprocessor to interface Agilent tools with the chip. More details on the experimental setup, programming, eraser, redesigned layout structure, half-select disturbance immunity, retention, and endurance characteristics are available in Ref.^52^. All eFlash memories are programmed to their targeted states at $V_{{{\text{WL}}}} = 1.5\,{\text{V}}, V_{{{\text{CG}}}} = 2.5\,{\text{V}}, V_{{{\text{BL}}}} = 1\,{\text{V}},V_{{{\text{SL}}}} = 0\,{\text{V}}, {\text{and}} V_{{{\text{EG}}}} = 0\,{\text{V}}$ and operated at the same biasing condition. Further, the devices are tuned one at a time by progressively increasing voltage pulses and using the write-verify algorithm. We have discussed the details of pulse amplitudes and durations in the programming phase in Ref.^52^.

As discussed in the main text, weight annealing is implemented by increasing the $V_{WL}$ from 0.7 to 1.5 V linearly and exponentially, which would exponentially and superexponentially increase the synaptic weights, respectively, since devices are operated in weak inversion (see Supplementary Materials S.7). Similar to the memristor-based circuit, we use the single device per synapse topology and compute each update in two cycles. The weights are mapped from software to hardware by using $I_{ij}^{T} = I_{{{\text{max}}}} \frac{{T_{ij} }}{{\left| {T_{{{\text{max}}}} } \right|}}$ and $I_{j}^{{\text{b}}} = I_{{{\text{max}}}} \frac{{T_{j}^{b} }}{{\left| {T_{max}^{b} } \right|}}$ in which $I_{{{\text{max}}}} = 1 \mu A$, $T_{{{\text{max}}}} = 2$ is the maximum absolute synaptic weight, and $T_{max}^{b} = 3.694$ is the maximum absolute bias.

## Supplementary information

Supplementary Information 1.

## Data Availability

The data that support the plots within this paper and are available from the corresponding author upon reasonable request.
