# Supplementary material for: Combinatorial optimization by weight annealing in memristive hopfield networks
Source: Sci Rep. 2021 Aug 12;11:16383. doi: 10.1038/s41598-020-78944-5 (PMC8361025; doi:10.1038/s41598-020-78944-5)
Supplement: Supplementary file 1 — Supplementary Information 1. [file 41598_2020_78944_MOESM1_ESM.pdf]

Combinatorial Optimization by Weight Annealing in Memristive Hopfield Networks Supplementary Materials

Z. Fahimi[1*#], M. R. Mahmoodi[1*#], H. Nili[1], Valentin Polishchuk[2], D. B. Strukov[1]
[1] UC Santa Barbara, Santa Barbara, CA 93106-9560, U.S.A. [2] Linkoping University, 60174 Norrkoping, Sweden
* Equal Contribution [#]{z.fahimi,mrmahmoodi}@ucsb.edu

Overview

Here, we provide supplementary details in support of the main paper. Section 1 briefly discusses the discrete-time Hopfield networks and annealing techniques. Section 2 formulates the optimization problems considered in this study. Section 3 expands upon the details of the 7-node graph partitioning problem. Section 4 and 5 are devoted to the additional graph partitioning, vertex cover, maximum clique, and maximum-independent set simulation results. Sections 6 and 7 include the relevant details of experiments performed with the two representative memory technologies. Section 8 and 9 discuss the performance prospects and the experimental setups, respectively.

1. Hopfield Neural Networks and Annealing Techniques

The focus of this paper is on discrete-time Hopfield neural networks (HNNs) [1,2]. The following Lyapunov energy function describes the dynamics of the network described by the update rule in Eq. 1 of the main text:

$$E = \frac{-1}{2}\sum_{j=1}^{N}\sum_{i=1,\neq j}^{N} T_{ij}U_i(t)U_j(t) - \sum_{j=1}^{N} T_j^b U_j(t). \tag{S1}$$

In the case of symmetric connections ($T_{ij} = T_{ji}$), zero self-feedback weights ($T_{ii} = 0$), and single neuron update at a time, the network always converges to a stable equilibrium point due to its gradient decedent dynamics. The change in the state of jth neuron at epoch t+1 results in ΔE given by

$$\Delta E = -\left(\sum_{i=1,\neq j}^{N} T_{ij}U_i(t) + T_j^{\text{b}}\right)\Delta U_j. \tag{S2}$$

Since ΔU_j and $\sum_{i=1,\neq j}^{N} T_{ij}U_i(t) + T_j^b$ have similar signs, $\Delta E \leq 0$, and since E is bounded, the equilibrium is a stable point. The search space is the interior of the N-dimensional hypercube, with minima located in the corners of it [3,4]. The most critical shortcoming of Hopfield networks (similar to the Ising model and other greedy and local search methods) is the presence of (many) local minima in their energy function. Although Eq. S2 guarantees the monotonic descendence of Lyapunov energy function, a local minima state may easily

trap the network. Therefore, these models, in general, may not find the global optimum solution. However, in many applications, finding an approximately "good" (and sometimes more energy efficient) solution in a reasonable time is preferred to finding the best solution slowly.

2. Combinatorial Optimization with Hopfield Networks

To implement a typical problem within the Hopfield model, we define an (intuitive) energy function, which its minima locate at the same points as the minima of the cost function (of the problem), and map the solution to the neuron states. The energy function is then compared with the generic Lyapunov function of HNN to find the synaptic weights. The inference involves initializing the neuron states or solution and then allowing the recurrent network to converge to a stable state that is interpreted as the ultimate solution. Several well-known combinatorial optimization problems are considered in our study as follows:

2a. Graph Partitioning

Graph bisection, the problem of minimizing the cutsize when partitioning a graph into two sections of nearly equal weight, finds applications in distributed computing and digital VLSI design flow. We define it over an n-vertex undirected graph $G = (V, E)$ in which w_i and e_{ij} $(1 \leq i, j \leq n)$ are the weights of the ith vertex and the edge between adjacent nodes i^{th} and j^{th}, respectively. The objective is to bisect the graph such that the sum of weight vertices assigned to each partition is equal while the sum of the disconnected edge weights is minimum. A single neuron (U_j) is attributed to each vertex (V_j). $U_j = 1$ determines that V_j belongs to partition 1 and $U_j = 0$ is otherwise. The cost function consists of two terms and is given by

$$E = \alpha \sum_{i=1}^{n} \sum_{j=1}^{n} e_{ij}(U_i + U_j - 2U_iU_j) + \sum_{i=1}^{n} \sum_{j=1}^{n} w_i w_j (1 - U_i - U_j + 2U_iU_j)$$

in which $\alpha = 0.5$ is a constant representing the relative importance of these two terms [5]. The first term is considered to minimize the weighted sum of the edges (which belong to each cut), and the second one to balance the sum of the node weights in two partitions. Rearranging and dropping the constant terms leads to

$$E = -\frac{1}{2} \sum_{i=1}^{n} \sum_{j=1,\neq i}^{n} \left(2e_{ij} - 4w_i w_j\right) U_i U_j - \sum_{i=1}^{n} U_i (2w_i \sum_{j=1}^{n} w_j - 2{w_i}^2 - \sum_{j=1}^{n} e_{ij})$$

which is very similar to the general energy function of Hopfield networks (Eq.S1) that is exploited to derive the weights as follows:

$$T_{ij} = 2e_{ij} - 4w_i w_j, \qquad T_i^{\mathrm{b}} = 2w_i \sum_{j=1}^{n} w_j - 2w_i^2 - \sum_{j=1}^{n} e_{ij}.$$

Note that -2w_i^2 term in bias weights is missing in Ref. [5].

2b. Minimum Weighted Vertex Cover Problem

The vertex cover is a fixed-parameter tractable and a central problem in parameterized complexity theory and one of 21 NP-complete problems in Karp's seminal work [6]. The adjacency matrix of G, $A = [a_{n\times n}]$, is defined by $a_{ij} = 1$ if there is an edge between i^{th} and j^{th} nodes of graph G, otherwise, $a_{ij} = 0$. The vertex cover C is a subset of G if all the edges of G are adjacent to at least one vertex in the set C. In minimum weighted vertex cover problems, the goal is to find the vertex cover with minimum cardinality (or size) whose total weight is also minimum [7]. Similar to the graph partitioning, one neuron is assigned per vertex. $U_i = 1$ determines that V_j is a part of the vertex cover. The cost function is defined by

$$E = \alpha \sum_{i=1}^{n} w_i U_i + \sum_{i=1}^{n} \sum_{j=1}^{n} a_{ij}(1 - U_i)(1 - U_j)$$

in which α is a constant representing the relative importance of these two terms. The first term is used to minimize the cardinality and total weight, and the second term ensures full coverage of the solution by penalizing the energy whenever there is an edge with endpoints not included in the cover. By dropping the constant terms and comparing them with the generalized energy function, we obtain the weights as

$$T_{ij} = -2a_{ij}, \qquad T_i^{\mathrm{b}} = 2\sum_{j=1}^{n} a_{ij} - \alpha w_i.$$

2c. Maximum Weight Independent Set Problem

In this problem, we are interested in finding the independent subset C of G with maximum total weight, which neither of its nodes is adjacent. The energy function is formulated by

$$E = -\alpha \sum_{i=1}^{n} w_i U_i + \sum_{i=1}^{n} \sum_{j=1}^{n} a_{ij} U_i U_j,$$

and all the parameters have the same meaning as before. The first term favors larger sets with larger weights and the second term penalizes the energy if there are adjacent nodes in C. Comparing it with the generic energy function leads to

$$T_{ij} = -2a_{ij}, \qquad {T_i}^{\mathrm{b}} = \alpha w_i.$$

2d. Maximum-Weight Clique Problem

A clique is a subset of G in which all nodes are adjacent to each other, and finding a clique with maximum cardinality and the maximum total weight is an NP-compete problem [6]. It finds many applications, e.g., in bioinformatics and analysis of random processes. We consider $U_i = 1$ when i^{th} node is a part of the maximum clique solution, and use the intuitive energy function

$$E = -\alpha \sum_{i=1}^{n} w_i U_i + \sum_{i=1}^{n} \sum_{j=1}^{n} (1 - a_{ij}) U_i U_j$$

in which the first term ensures that the larger the sum of weights in the maximum clique, the lower the energy, and the second term penalizes the energy when the solution includes non-adjacent nodes. We compare it with the general Lyapunov energy function of the Hopfield model to find the weights:

$$T_{ij} = 2(a_{ij} - 1), \qquad {T_i}^{\mathrm{b}} = \alpha w_i.$$

3. 7-node graph partitioning problem parameters

An arbitrary graph G=(V, E) with the following elements are used (all numbers are rounded to 2 decimal points for clarity):

$$V = \begin{pmatrix} 0 & 84.17 & 3.88 & 50.64 & 27.53 & 30.86 & 13.21 \\ 84.17 & 0 & 16.31 & 81.04 & 65.84 & 45.65 & 14.21 \\ 3.88 & 16.31 & 0 & 94.10 & 92.18 & 61.67 & 9.09 \\ 50.64 & 81.04 & 94.10 & 0 & 47.69 & 69.23 & 65.51 \\ 27.53 & 65.84 & 92.18 & 47.69 & 0 & 38.24 & 28.12 \\ 30.86 & 45.65 & 61.67 & 69.23 & 38.24 & 0 & 82.18 \\ 13.21 & 14.21 & 9.09 & 65.51 & 28.12 & 82.18 & 0 \end{pmatrix}, E = \begin{pmatrix} 5.64 \\ 3.80 \\ 4.79 \\ 3.42 \\ 8.43 \\ 9.65 \\ 5.01 \end{pmatrix}$$

Then, we employ the energy equations to find the weight matrices:

$$T = \begin{pmatrix} 0 & -100.33 & 23.96 & -100.33 & -135.30 & -156.13 & -86.65 \\ 82.58 & 0 & -40.19 & 110.00 & 3.44 & -55.44 & -47.74 \\ -100.33 & -40.19 & 0 & 122.55 & 22.71 & -61.63 & -77.83 \\ 23.96 & 110.00 & 122.55 & 0 & -20.24 & 6.15 & 62.34 \\ -135.30 & 3.44 & 22.71 & -20.24 & 0 & -249.28 & -112.86 \\ -156.13 & -55.44 & -61.63 & 6.15 & -249.28 & 0 & -29.15 \\ -86.65 & -47.74 & -77.83 & 62.34 & -112.86 & -29.15 & 0 \end{pmatrix}, T^{\mathrm{b}} = \begin{pmatrix} 185.93 \\ -26.32 \\ 67.35 \\ -152.39 \\ 245.76 \\ 272.75 \\ 145.95 \end{pmatrix}$$

The runtime change in synaptic weights is shown in Fig. S1a. The weights are slowly deformed, and the network tends to remain in the transitory ground state during the runtime. The results in Fig. 1Sb compare exponential weight annealing with the baseline on all possible runs. The annealing schedule is $\tau = 40$.

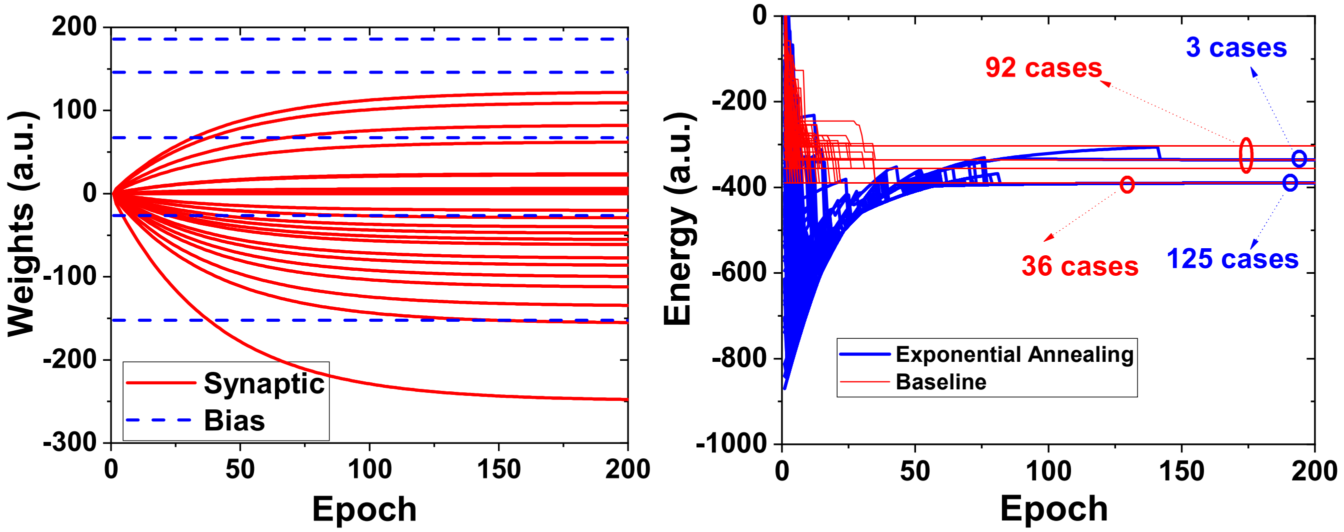

Fig. 1S: (a) The change of synaptic weights for the weight annealing technique when solving the 7-node graph partitioning problem. (b) Comparison between the results obtained by exponential weight annealing versus baseline for all 128 runs.

4. Additional data on graph partitioning

We have conducted three sets of extensive simulations on weighted graph partitioning. For all configurations, the vertex and edge weights are selected randomly in the ranges of [2:N] and [0:20], respectively. The impact of scaling the problem size is studied for a fixed number of epochs in the main text (Fig. 1d). Here, the simulation results are provided for the case when we scale up the number of epochs with the problem size. N_{EP} is exponentially increased with respect to the problem size in Fig. 2S. The solution quality of weight annealing

is better than simulated annealing for N>10, as shown in Fig. 2S. Table I shows the parameters used in Figs. 1d, 1e, and 1f (of the main text), and Fig. 2S of supplementary materials.

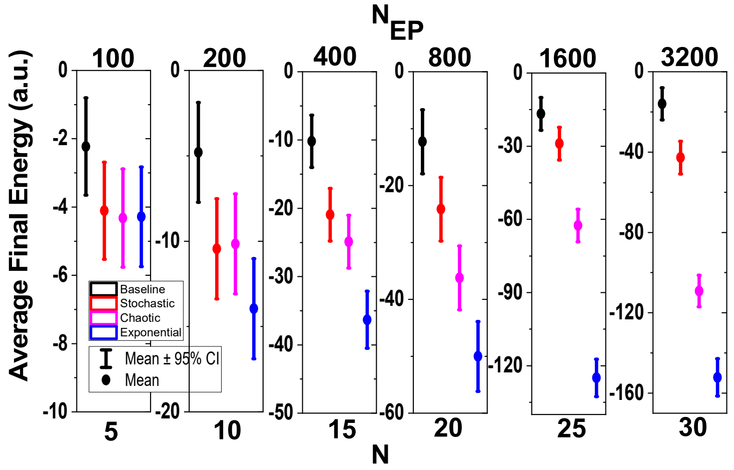

Fig. 2S: Extended graph partitioning simulations: The result of increasing the computational time (N_{EP}) with respect to the graph size (N). Note that, in this figure, the average energy is added by a constant for better clarity.

Table I: The parameters used in the graph-partitioning simulations

	N	Epoch Number	Case numbers	Annealing Schedule		
				Stochastic	Chaotic	Exponential
Fig.1d	5	300	32	50	50	60
	10	300	1000	60	500	60
	15	300	10000	70	1e3	60
	20	300	10000	70	5e3	60
	25	300	10000	100	9e3	60
	30	300	10000	300	2e4	60
	40	300	10000	500	1e5	60
	50	300	10000	1000	2e5	60
Fig.1f	25	100	10000	150	1e3	20
	25	200	10000	200	4e3	40
	25	400	10000	1e3	1e4	80
	25	800	10000	5e3	1e5	160
	25	1600	10000	1e4	1e6	320
	25	3200	10000	5e4	1e7	640
	25	6400	10000	1e5	1e8	1280
	25	12800	10000	1e6	1e9	2560
Fig.2S	5	100	32	30	30	20
	10	200	1000	60	60	40
	15	400	10000	90	3e3	80
	20	800	10000	200	7e3	160
	25	1600	10000	700	1e5	320

	30	3200	10000	2e3	1e6	640
	40	6400	10000	1e4	1e7	1280
	50	12800	10000	1e5	1e8	2560

5. Simulation results on minimum weighted vertex cover, maximum weighted clique, and maximum weighted independent set problems

Similar sets of simulations are performed for three other combinatorial optimization problems as well. Note that simulation parameters (N and N_{EP}) are the same as those for the graph partitioning problem (Table. I), while the annealing schedule might be slightly different since they are manually optimized for each problem type/size. Figs. 3S, 4S, and 5S show the results of the maximum independent set, maximum clique, and minimum vertex cover problems. Similar trends are observed, and weight annealing outperforms other annealing techniques. For the vertex cover, we notice that the weight annealing fails to find the exact solution (i.e., the global optimum) for few large graph configurations. Note that weight annealing is still better or on par with chaotic annealing on average and outperforms simulated annealing in terms of response quality (Top-5 and average energy). We believe that some spurious states generated during the annealing are responsible for those rare underperforming cases.

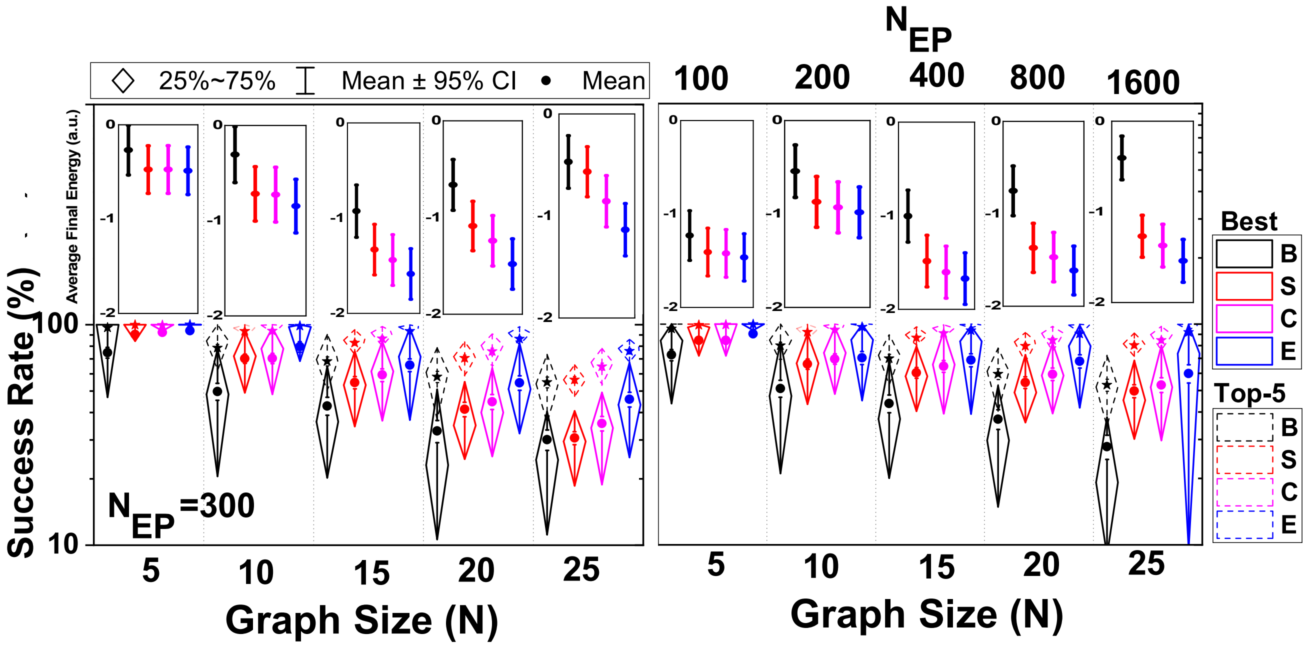

Fig. 3S: Simulation results on 200 random configurations of the maximum weighted independent set problem with various sizes: (a) the success rate and (the average final energy in the inset) for different sizes and fixed computational time (N_{EP}=300), (b) the success rate and (the average final energy in the inset) for different sizes with a varying number of epochs. Note that, in each figure, the actual average energy is added by a constant for better clarity.

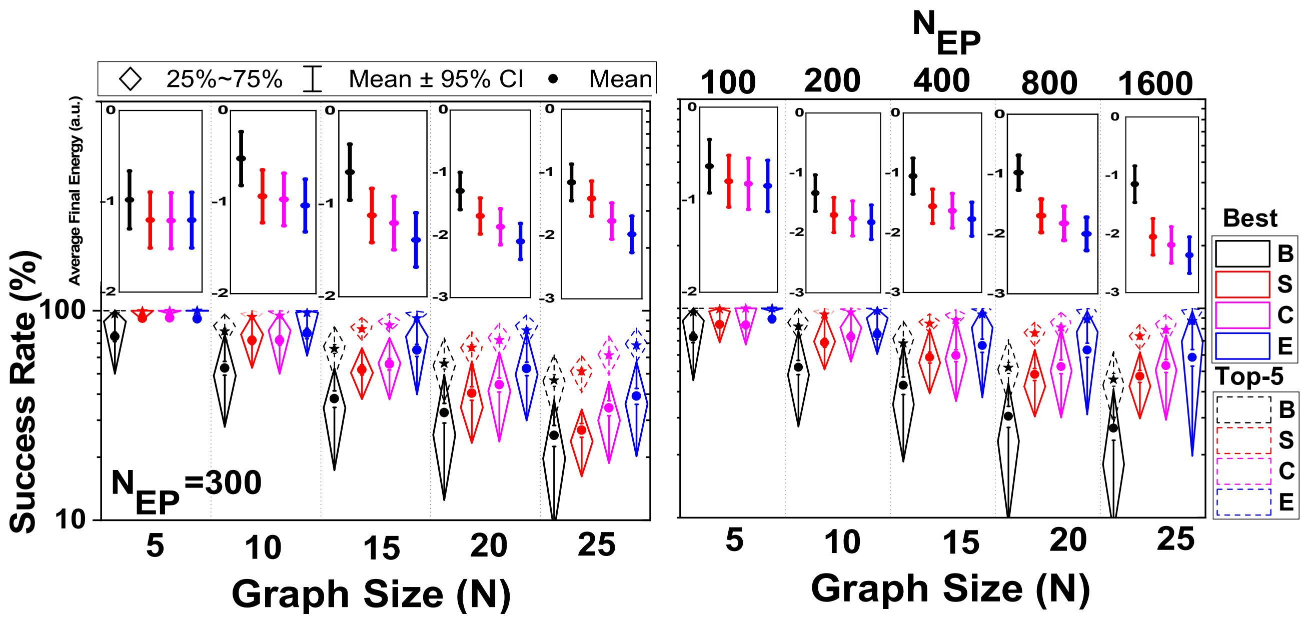

Fig. 4S: Simulation results on 200 random configurations of the maximum weighted clique problem with various sizes: (a) the success rate and (the average final energy in the inset) for different sizes and fixed computational time (N_{EP}=300), (b) the success rate and (the average final energy in the inset) for different sizes with a varying number of epochs. Note that, in each figure, the actual average energy is added by a constant for better clarity.

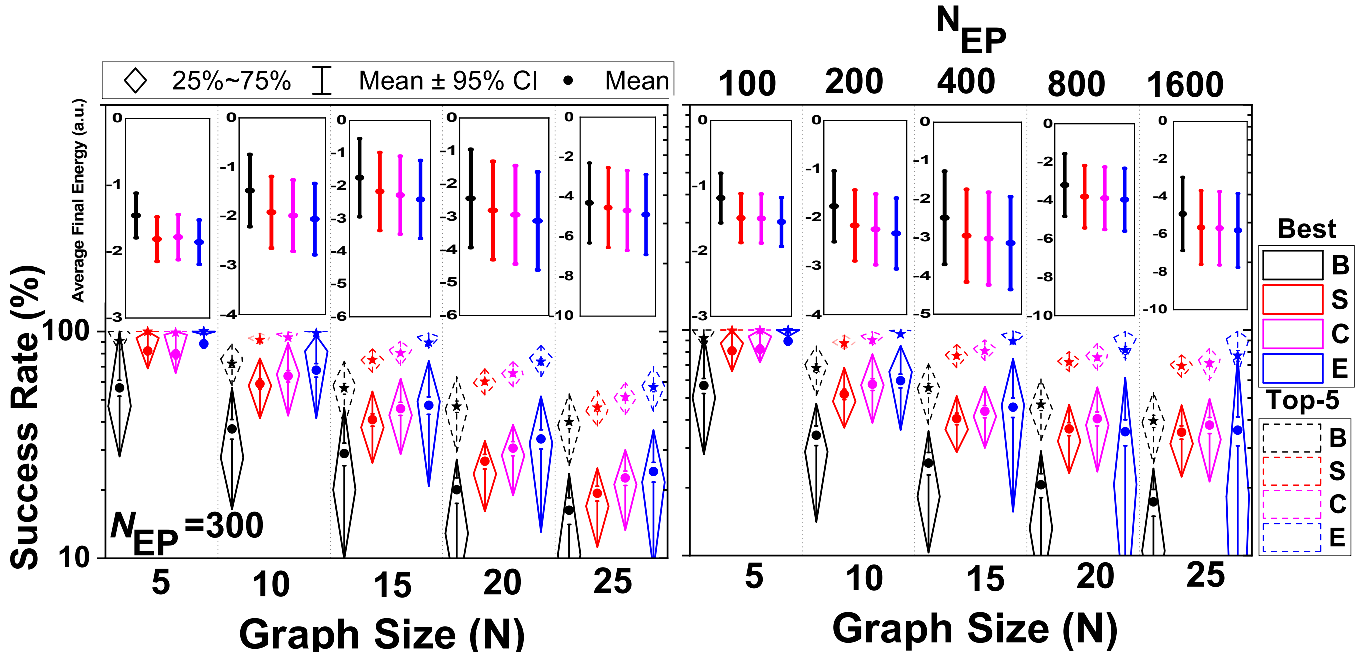

Fig. 5S: Simulation results on 200 random configurations of the minimum weighted vertex cover with various sizes: (a) the success rate and (the average final energy in the inset) for different sizes and fixed computational time (N_{EP}=300), (b) the success rate and (the average final energy in the inset) for different sizes with a varying number of epochs. Note that, in each figure, the actual average energy is added by a constant for better clarity.

6. Additional details on the 13-node graph partitioning experiment

A random graph $G=(V, E)$ with the following analog weights are used in the memristor-based hardware implementations of weight annealing:

$$V=\begin{pmatrix} 0 & 11 & 6 & 4 & 6 & 1 & 1 & 16 & 18 & 25 & 9 & 4 & 5 \\ 11 & 0 & 12 & 29 & 30 & 2 & 16 & 10 & 14 & 5 & 14 & 9 & 17 \\ 6 & 12 & 0 & 30 & 15 & 3 & 25 & 6 & 18 & 0 & 1 & 5 & 14 \\ 4 & 29 & 30 & 0 & 14 & 8 & 3 & 12 & 8 & 3 & 2 & 19 & 5 \\ 6 & 30 & 15 & 14 & 0 & 7 & 16 & 18 & 7 & 8 & 5 & 13 & 5 \\ 1 & 2 & 3 & 8 & 7 & 0 & 5 & 14 & 1 & 28 & 16 & 30 & 7 \\ 1 & 16 & 25 & 3 & 16 & 5 & 0 & 15 & 14 & 5 & 3 & 4 & 0 \\ 16 & 10 & 6 & 12 & 18 & 14 & 14 & 0 & 5 & 13 & 18 & 20 & 13 \\ 18 & 14 & 18 & 8 & 7 & 1 & 14 & 5 & 0 & 16 & 13 & 19 & 14 \\ 25 & 5 & 0 & 3 & 8 & 28 & 5 & 13 & 16 & 0 & 18 & 18 & 17 \\ 9 & 14 & 1 & 2 & 5 & 16 & 3 & 18 & 13 & 18 & 0 & 10 & 2 \\ 4 & 9 & 5 & 19 & 13 & 30 & 4 & 20 & 19 & 18 & 10 & 0 & 14 \\ 5 & 17 & 14 & 5 & 5 & 7 & 0 & 13 & 14 & 17 & 2 & 14 & 0 \end{pmatrix}, E=\begin{pmatrix} 10 \\ 5 \\ 4 \\ 6 \\ 3 \\ 6 \\ 5 \\ 13 \\ 5 \\ 14 \\ 10 \\ 5 \end{pmatrix}.$$

Then, the procedure described in section 2 is utilized to find the synaptic weights and bias matrices of the HNN model:

$$T=-\begin{pmatrix} 0 & 178 & 148 & 192 & 228 & 118 & 238 & 168 & 484 & 150 & 542 & 392 & 190 \\ 178 & 0 & 56 & 42 & 42 & 56 & 88 & 80 & 232 & 90 & 252 & 182 & 66 \\ 148 & 56 & 0 & 20 & 20 & 42 & 46 & 68 & 172 & 80 & 222 & 150 & 52 \\ 192 & 42 & 20 & 0 & 92 & 44 & 114 & 76 & 244 & 94 & 276 & 162 & 90 \\ 228 & 60 & 66 & 92 & 0 & 58 & 112 & 84 & 298 & 104 & 326 & 214 & 110 \\ 118 & 56 & 42 & 44 & 58 & 0 & 62 & 32 & 154 & 4 & 136 & 60 & 46 \\ 238 & 88 & 46 & 114 & 112 & 62 & 0 & 90 & 284 & 110 & 330 & 232 & 120 \\ 168 & 80 & 68 & 76 & 84 & 32 & 90 & 0 & 250 & 74 & 244 & 160 & 74 \\ 484 & 232 & 172 & 244 & 298 & 154 & 284 & 250 & 0 & 228 & 702 & 482 & 232 \\ 150 & 90 & 80 & 94 & 104 & 4 & 110 & 74 & 228 & 0 & 244 & 164 & 66 \\ 542 & 252 & 222 & 276 & 326 & 136 & 330 & 244 & 702 & 244 & 0 & 540 & 276 \\ 392 & 182 & 150 & 162 & 214 & 60 & 232 & 160 & 482 & 164 & 540 & 0 & 172 \\ 190 & 66 & 52 & 90 & 110 & 46 & 120 & 74 & 232 & 66 & 276 & 172 & 0 \end{pmatrix}, T^b=\begin{pmatrix} 1514 \\ 691 \\ 561 \\ 723 \\ 876 \\ 406 \\ 913 \\ 700 \\ 1881 \\ 704 \\ 2045 \\ 1455 \\ 747 \end{pmatrix}.$$

To realize weight annealing within our memristive crossbars, we encode the normalized weights ($T_{ij} < 0$ and $T_j^b > 0$) to device conductances by using $g_{ij} = G_{\max}(T_{ij}/|W^{\max}|)$ and $g_j^b = G_{\max}(T_j^b/|W^{\max}|)$ where $|W^{\max}| = \max\{|T|,|T^b|\}$, where $G_{\max}$ is a predetermined value. Memristive crossbars inherently implement the dot-product using Ohm and Kirchhoff's laws, i.e.,

$$V(t+1) = \mathrm{f}\left(\sum_{i=1}^{N} g_{ij}V_i(t) + g_j^b V^{\mathrm{ap}}\right). \tag{S3}$$

Note that f(.) is the binary activation function realized by a comparator in peripheral circuits. In our experimental setup, straightforward peripheral functionalities such as current sensing and binary activation

function are emulated by Agilent characterization tools. In exponential weight annealing, $V_i(t) = V_i^{\mathrm{ap}}(t)(1 - \mathrm{e}^{\frac{-t}{\tau}})$ is the applied voltage to the ith input and $V_i^{\mathrm{ap}}(t)$ is either 0 or V_{ap}, based on the neuron state. Given that, we rewrite Eq. S3 as

$$V(t+1) = \mathrm{f}\left(\frac{V^{\mathrm{ap}} G_{\max}}{|W^{\max}|} \sum_{i=1}^{N} T_{ij}(V_i(t)\,/V^{\mathrm{ap}}) + T_j^{\mathrm{b}}\right)$$

$$= \mathrm{f}\left(\frac{V^{\mathrm{ap}} G_{\max}}{|W^{\max}|} \sum_{i=1}^{N} T_{ij}(V_i^{\mathrm{ap}}(t)(1 - e^{\frac{-t}{\tau}})\,/V^{\mathrm{ap}}) + T_j^{b}\right)$$

$$= \mathrm{f}\left(\frac{V^{\mathrm{ap}} G_{\max}}{|W^{\max}|} \sum_{i=1}^{N} w_{ij}(t)(V_i^{ap}(t)/V^{\mathrm{ap}}) + T_j^{b}\right), \quad \text{(S4)}$$

which is essentially the hardware implementation of Eq. 1 of the main text.

7. Additional details the 7-node maximum weight independent set experiment

For the eFlash-based experimental demonstration, a graph with the following adjacency matrix and nodal weights are assumed:

$$A = \begin{pmatrix} 0 & 1 & 1 & 0 & 1 & 1 & 1 \\ 1 & 0 & 1 & 1 & 1 & 1 & 1 \\ 1 & 1 & 0 & 1 & 1 & 0 & 0 \\ 0 & 1 & 1 & 0 & 1 & 1 & 1 \\ 1 & 1 & 1 & 0 & 0 & 1 & 1 \\ 1 & 1 & 0 & 1 & 1 & 0 & 1 \\ 1 & 1 & 0 & 1 & 1 & 1 & 0 \end{pmatrix}, E = \begin{pmatrix} 6.40 \\ 7.38 \\ 5.05 \\ 1.21 \\ 3.43 \\ 2.02 \\ 6.09 \end{pmatrix}$$

Section S2 discusses the equations for solving the 7-node maximum weight independent set problem. Accordingly, the synaptic weights are found by assuming $\alpha = 0.5$:

$$T = -\begin{pmatrix} 0 & 2 & 2 & 0 & 2 & 2 & 2 \\ 2 & 0 & 2 & 2 & 2 & 2 & 2 \\ 2 & 2 & 0 & 2 & 2 & 0 & 0 \\ 0 & 2 & 2 & 0 & 2 & 2 & 2 \\ 2 & 2 & 2 & 0 & 0 & 2 & 2 \\ 2 & 2 & 0 & 2 & 2 & 0 & 2 \\ 2 & 2 & 0 & 2 & 2 & 2 & 0 \end{pmatrix}, T^b = \begin{pmatrix} 3.20 \\ 3.69 \\ 2.52 \\ 0.60 \\ 1.71 \\ 1.01 \\ 3.04 \end{pmatrix}$$

The embedded flash memory array consists of supercells (Fig. 6S), and we can tune each floating-gate cell to a desired current value at nominal biasing conditions. Neglecting the drain-source voltage, $I_{\mathrm{DS}} \approx$

$I_0 e^{\frac{V_{\mathrm{WL}}-V_{\mathrm{th}}}{nV_{\mathrm{th}}}} \approx \theta_0 I_0 e^{\frac{V_{\mathrm{WL}}}{nV_{\mathrm{th}}}}$ where θ_0 is the effective weight of the devices, and other parameters have their usual meaning. We define $I_{ij}(V_{\mathrm{WL}}) = \theta_{ij} I_0 e^{\frac{V_{WL}}{nV_{\mathrm{th}}}}$ and $I_j^b = \theta_j^b I_0 e^{\frac{V_{\mathrm{ap}}}{nV_{\mathrm{th}}}}$. During tunning, we make sure that $I_{ij}(V^{\mathrm{ap}}) = \frac{I_{\max} T_{ij}}{|W^{\max}|}$ and $I_j^b = \frac{I_{\max} T_j^b}{|W^{\max}|}$ where $|W^{\max}| = \max\{|T|,|T^b|\}$, and $I_{\max}$ is a predetermined value. Hence,

$$\theta_{ij} = \frac{I_{\max} T_{ij}}{|W^{\max}| I_0} e^{\frac{-V^{\mathrm{ap}}}{nV_{\mathrm{th}}}}, \qquad \theta_j^b = \frac{I_{\max} T_j^b}{|W^{\max}| I_0} e^{\frac{-V^{\mathrm{ap}}}{nV_{\mathrm{th}}}} \tag{S5}$$

The update rule for the jth neuron is given by

$$V(t+1) = \mathrm{f}\left(\sum_{i=1}^{N} I_{ij}(V_i(t)) + I_j^b\right) \tag{S6}$$

and $V_i(t)$ is the gate-applied voltage to the ith input terminal at iteration t. It is effortless to show that S6 performs the dot-product of (two-state) input voltage vector and (analog) weights. Note that we apply the digital inputs ($V_{\mathrm{WL}} = 0$ V corresponds to ~0 synaptic current and $V_{\mathrm{WL}} = V^{\mathrm{ap}} = 1.5\ V$ corresponds to the 'on' current for the baseline operation) in the gate-line direction and read the summed currents from the bitlines, (e.g., using a transimpedance amplifier or a current conveyor) to $V_{BL} = 1\ V$.

For the exponential weight annealing, we substitute I_{ij} and I_j^{b} in S6 using S5 and obtain

$$V(t+1) = \mathrm{f}\left(\sum_{i=1}^{N} \frac{I_{\max} T_{ij}}{|W^{\max}| I_0} e^{\frac{-V^{\mathrm{ap}}}{nV_{\mathrm{th}}}} I_0 e^{\frac{V_i(t)}{nV_{\mathrm{th}}}} + \frac{I_{\max} T_j^{\mathrm{b}}}{|W^{\max}| I_0} e^{\frac{-V^{\mathrm{ap}}}{nV_{\mathrm{th}}}} I_0 e^{\frac{V_{\mathrm{ap}}}{nV_{\mathrm{th}}}}\right) =$$

$$= \mathrm{f}\left(\frac{I_{\max}}{|W^{\max}|} \sum_{i=1}^{N} T_{ij} e^{\frac{V_i(t)-V^{\mathrm{ap}}}{nV_{\mathrm{th}}}} + T_j^{\mathrm{b}}\right)$$

and $V_i(t) = \frac{V_i^{\mathrm{ap}}(t)t}{\tau}$ (τ is the annealing schedule) for the semi-exponential learning (it is "semi" since the devices have nonlinearities in their *I-V*) and $V_i(t) = V_i^{\mathrm{ap}}(t)(1 - e^{\frac{-t}{\tau}})$ for the super-exponential learning. Fig. 7Sa shows the weight change for the former case, i.e., linear voltage scaling with $\tau = 500$, and the main synaptic weights grow semi-exponentially. Also, since the currents are extremely small for $V_{WL} < 0.7$, the

ground state of the system remains constant for a significant period. We prevent this by starting from $V_{\mathrm{WL},0} = 0.7$ and govern the annealing by

$$V_i(t) = \frac{(V_i^{ap}(t) - V_{WL,0})t}{\tau} + V_{WL,0}.$$

Fig. 7Sb shows the experimental results on the impact of the annealing schedule and linear versus exponential voltage scaling. We obtain a better solution with slower annealing, particularly with the linear scaling in which the trend is much more apparent. The slowest linear scaling annealing, i.e., when $\tau_{\mathrm{lin}} = 500$ leads to the best solution.

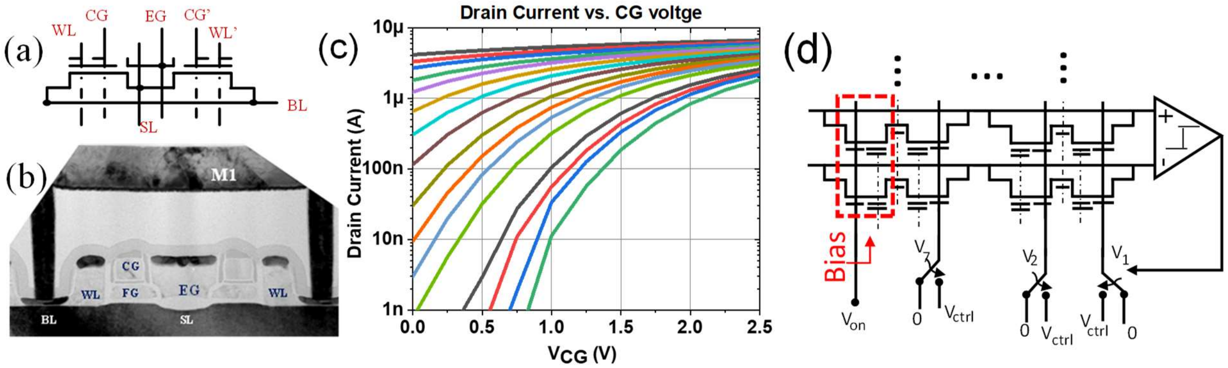

Fig. 6S: (a) The schematic of SST's eFlash supercell consisting of 2 analog-grade floating-gate cells and (b) its TEM image. (c) The weak inversion *I-V* characteristics of a flash cell programmed with 1% accuracy. (d) The schematic of a single channel recurrent neuro-optimizer. For clarity, we show the feedback signal only from the first channel.

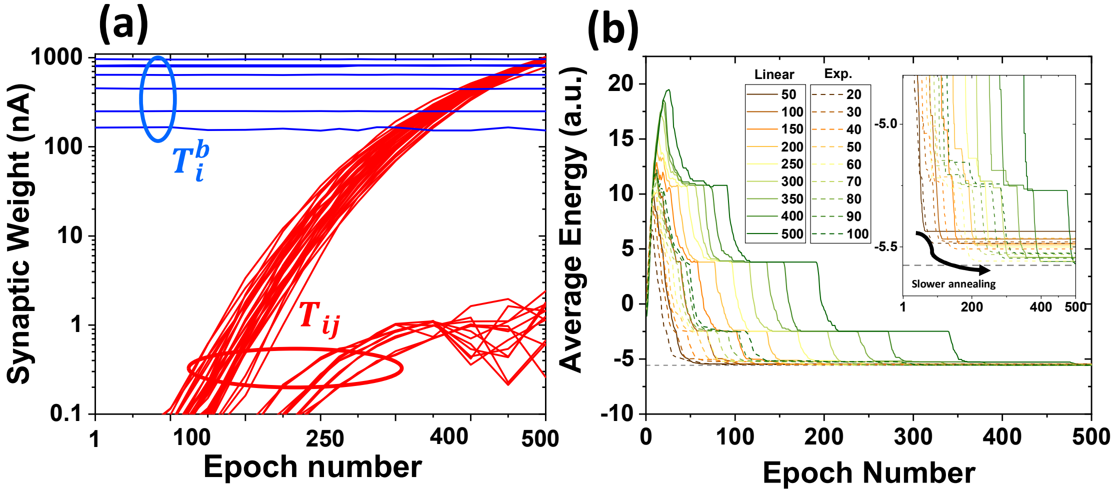

Fig. 7S: Extended experimental results of the 7-node maximum weighted independent set problem: (a) weight evolution during weight annealing (linear voltage scaling), (b) the impact of changing the annealing schedule (τ) for both cases of linear and exponential voltage scaling.

8. The performance of the proposed circuit

We design the peripheral circuits (amplifiers, current reference, and dynamic comparator) in Global Foundries 55 nm CMOS technology to determine the power, area, and speed of the circuit. 3-stage recycling folded cascode amplifiers are considered for designing the transimpedance amplifiers in the presynaptic and postsynaptic circuits and use the strongARM latch to develop the dynamic comparator. Flash memories have a large output impedance in weak inversion. This allows us to use faster and more compact circuits, e.g., current conveyors, for designing the readout circuit (see [25]). We assume 130×64 arrays, which can implement a full-differential 64×64 Hopfield network. In our estimation, we also consider the area of tuning circuits, analog switches, and decoders and exclude clocking and IO circuits as they can be shared with other system-on-chip modules. The circuits are designed targeting 2 ns settling time in dot-product operation and 0.5 ns sampling time in the comparator (hence, ~2.5 ns/update) in the typical corner. Such design parameters lead to ~0.33 pJ/update, ~3,038 μm^2 active area with 4 F^2 memristors, assuming 1 µs annealing schedule (2,000 epochs per run). For the eFlash technology, we obtain 0.09 pJ/update and ~10,992 μm^2 active area with ~110 F^2 redesigned eFlash memories, assuming 1 µs annealing schedule. These preliminary data suggest that our approach could potentially be $10^2\times$ faster and $10^5\times$ more energy efficient as compared to the most efficient conventional methods based on graphics processor units on the same task.

9. Experimental Setups

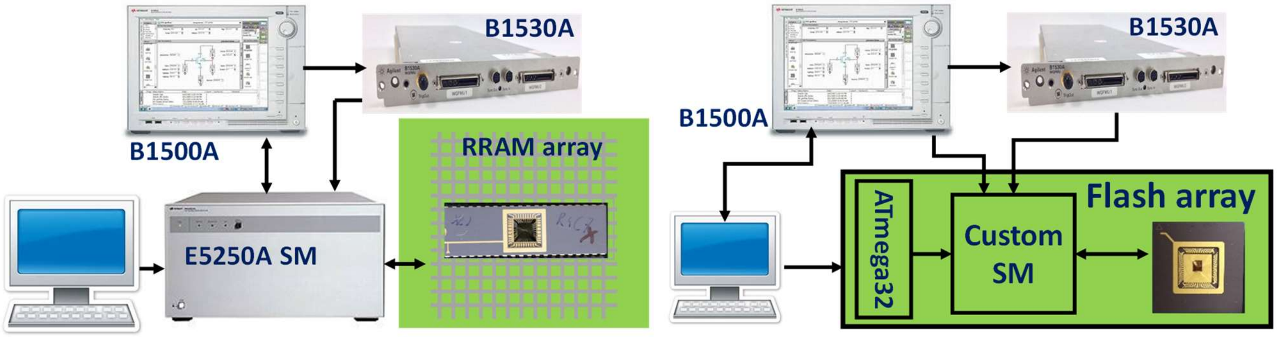

Fig. 8S: The Experimental setup for (a) the 13-node graph partitioning implemented with memristive devices and (b) the 7-node maximum weighted independent set realized with an eFlash memory array. The experimental setups comprise of a personal computer to control parameter analyzer B1500A, arbitrary waveform generator B1530A, and a switch matrix (a low-leakage Agilent E5250A in (a) and a custom circuit for (b)). Both memristive crossbar and flash array are mounted on custom host circuit board and connected to their corresponding switch matrices.

References

[1] Hopfield, John J., and David W. Tank. ""Neural" computation of decisions in optimization problems." Biological cybernetics 52.3 (1985): 141-152.

[2] Hopfield, John J. "Neural networks and physical systems with emergent collective computational abilities." Proceedings of the national academy of sciences 79.8 (1982): 2554-2558

[3] Tank, Df, and J. J. Hopfield. "Simple' neural 'optimization networks: An A/D converter, signal decision circuit, and a linear programming circuit." IEEE transactions on circuits and systems 33.5 (1986): 533-541.

[4] Joya, Gonzalo, M. A. Atencia, and Francisco Sandoval. "Hopfield neural networks for optimization: study of the different dynamics." Neurocomputing 43.1-4 (2002): 219-237.

[5] J. Ramanujam and P. Sadayappan, "Mapping combinatorial optimization problems onto neural networks", *Information Science*, vol. 82, pp. 239-255, 1995.

[6] R. M. Karp, "Reducibility among combinatorial problems", *Complexity of computer computations, Springer*, Boston, MA, pp. 85-103, 1972.

[7] M. N. Syed, and P. M. Pardalos, "Neural network models in combinatorial optimization", *Handbook of Combinatorial Optimization*, pp. 2027-2093, 2013.

[8] M. R. Mahmoodi, and D. Strukov. "An ultra-low energy internally analog, externally digital vector-matrix multiplier based on NOR flash memory technology." *Proceedings of the 55th Annual Design Automation Conference*. 2018.
